# The “Culture” of Organs: A Holistic Theory on the Origins of the Cancer Tissue Environment

**DOI:** 10.3390/life14121622

**Published:** 2024-12-07

**Authors:** Robert D. Rehnke

**Affiliations:** Private Practice of Plastic Surgery, Saint Petersburg, FL 33710, USA; robertrehnke@me.com

**Keywords:** cancer, epithelial parenchyma, interstitium, developmental autopoiesis, breast cancer, reticular fascial network, mesenchyme, intra-organic spaces, ontogenesis, cancer tissue environment

## Abstract

For over a century, the somatic gene mutation theory of cancer has been a scientific orthodoxy. The recent failures of causal explanations using this theory and the lack of significant progress in addressing the cancer problem medically have led to a new competition of ideas about just what cancer is. This essay presents an alternative view of cancer as a developmental process gone wrong. More specifically, cancer is a breakdown in the autopoietic process of organ maintenance and the multicellular coordination of tissues. Breast cancer is viewed through a systems science perspective as an example of the importance of framing one’s theoretical assumptions before making empirical judgments. Finally, a new understanding of the histoarchitecture of the interstitium is presented as a first principle of cancer: a process of cells coming from cells, invading the space between cells.

## 1. Introduction

“He deals the cards as a meditation, … to find the answer, the sacred geometry of chance, the hidden law of a probable outcome, the numbers lead a dance…”

Sting, “The Shape of My Heart”.

“A little neglect may breed great mischief… For the want of a nail the shoe was lost; for the want of a shoe the horse was lost; and for want of a horse the rider was lost… For the want of a battle the kingdom was lost, And all for the want of a horseshoe-nail”. 

Benjamin Franklin, *Poor Richard’s Almanac*.

I have been fascinated by biology, especially human biology, my whole life. As a doctor, I have dedicated myself to the study of health and disease. I have been interested in a particular organ’s health—the breast. Most of my professional thinking, as a general surgeon then a plastic surgeon, has focused on breast cancer and its treatment. Recent paradigm shifts in biology, evolution, and cancer research have led to the realization that life and disease are too complicated to be solely explained at the molecular and cellular level [1]. Life is a competition for free energy in the environment, needed for a limited time to reduce entropy locally, to stay alive, so that we can be sure to pass life on. Cancer too must be understood at more than the molecular and cellular level; it must be thought about at the tissue, organ, and organismal level [2]. The Somatic Gene Theory’s failure to explain the discovery of an ever-increasingly complex set of characteristics, of the spectrum of neoplasia, has opened the door for questioning this orthodoxy. Multiple alternative theories of the causation of cancer are now being debated [3,4,5,6,7,8].

## 2. Breast Cancer as a Developmental Disease

Breast cancer is thought to be a developmental disease of the breast organ [9]. That is, breast cancer is a fundamental breakdown of the coordinating limiting factors of tissues: cells and the extracellular protein matrix (ECM), the so-called tumor microenvironment (TME). Most of the important information presented in this paper was published in the 1990s. However, not until just recently have cytologists, a term first coined in 1962 by Smithers [10], who have led a publication filibuster for over 60 years, yielded the floor to the organismists. Hanahan’s 2024 publication raised the number of *hallmarks* to such a level that the Somatic Gene Theory has spun out of orbit [11]. Weinberg’s solo publication admits it is time for biologists studying cancer to go back to the drawing board [12]. 

The cells and proteins, which first come together two months after conception, make the breast gland which matures into the adult form at puberty [13]. The hormonal milieu of pregnancy sets into motion a spring-time season of the female breast tissue that is a recapitulation of embryonic lactiferous epithelial development. The breast gland fulfills its destiny by realizing the production of milk that sustains the infant until it is mature enough to fend for itself. When man lived in small hunter-gatherer bands, at the origin of our species, successful breast feeding was essential to leaving behind progeny. Prior to the epigenetic development of larger society, which evolved with the start of agriculture and animal husbandry, followed by complex civilizations with wet nurses or substitutes like cow’s milk and ultimately grocery store formulas, the failure of the mother’s breasts was an existential threat to the survival of the human species. Perhaps it was the Neanderthal breast that was inferior. It is therefore little wonder that human *culture* would put such a high priority on the health and maintenance of the breast *organ*. If a woman’s years of fertility are limited and if bad luck has led to barrenness, then the last successful ovulation which leads to impregnation must lead to successful milk production by the breast organs for the offspring to survive infancy. But what rules regulate the complex set of interactions involved in the above scenario? Put another way, in the absence of human societal culture, the culture of organs must prevail for the preservation of the species.

## 3. Living Anatomy of Organs

In the late 1930s, just before the outbreak of war in Europe, an important scientific study was taking place within biology. The French surgeon, Alexis Carrel, awarded the Nobel Prize for the discovery of the technique for vascular anastomosis, had turned his attention to organ transplantation and tissue engineering [14]. In 1938, he and his famous friend, Charles Lindbergh, published “The Culture of Organs”. The removal of whole organs from experimental animals for physiological study would require that Lindbergh, who entered medical research after having watched his sister-in-law die prematurely from a heart condition, invent a perfusion pump to keep organs alive and functioning outside the body [15]. Carrel, who in the early 1900s was a pioneer in the burgeoning field of cell culture, had devised the fluid medium that would be pumped. But it was Alexis’ vision of the importance of studying the *living* anatomy of *whole* organs and not just dissected dead parts that set the scientific project apart. Carrel died of natural causes shortly after World War II, thus reducing his influence on biologic thought in the second half of the 20th century. The 1950s and 1960s would usher in a 70-year reign of cell biology, genetics, and the study of cancer cells in flasks—not perfused organs. Only just now are we fully appreciating what a mistake this wrong turn in history was. The following paragraphs are taken from the first few pages of chapter one of Carrel and Lindbergh’s 1938 book, *The Culture of Organs* [16].

“The concept of tissue and organs, as taught by classical anatomy, are simple and convenient. But, like all abstractions, they are an incomplete expression of reality. They have shown their usefulness in providing a basis for physiology and pathology… It does not unveil the mechanism of most commonly observed phenomena, such as inflammation of a tissue, growth of a tumor, cicatrization of a wound. Living organs differ profoundly from organs removed by dissection from the body, because tissues and circulating blood constitutes an indivisible whole… A cell is bound to medium (lymph filling the intra-organic spaces) strictly as nucleus to cytoplasm”. 

“This medium is secreted by tissues and organs and, in turn, regulates their activity… The structure and functions of an organ rest simultaneously on its hereditary properties, on its previous history, and on the state of its humors… These chemicals (components of blood plasma) are indispensable to the constitution of each organ as are epithelial and connective cells and their framework… Cells and medium are one”. 

“An organ separated from the body by dissection is rendered timeless and functionless. Structure, time, and function, are only aspects of the wholeness of living organisms. … An organ is essentially an enduring thing. It is a movement, a ceaseless change within the frame of an identity. The duration of an organism is equivalent to those chemical changes of tissue and blood plasma that express themselves in growth, maturity, old age. The rigidity and immobility of their appearance are illusions, because they flow into physical time at the same rhythm as the observer… Physiological time is merely the fourth dimension of the living organism”. 

## 4. The New Biology

It is this ethos that typifies the new era we enter, in what Phillip Ball has called the “New Biology” [17]. It is a break from the old biology: reductionist, simple narratives about parts built of smaller and smaller parts, which function linearly with predictable outcomes. The old biology is built on a Newtonian world, but the new biology is a quantum world which is complex and full of non-linear systems that have stochastic outcomes, are built of emergent properties, and quite possibly… show biological entanglement [18]. This is the world of the human embryo at eight weeks, when the breast anlage emerges [19]. The embodied truths of ontogenesis are essential to understanding the origins of cancer. Therefore, future advances in cancer research will require us to return to the beginning of the story of tissue and organ development.

## 5. Spatial Opportunity and Metabolic Occasion 

Erich Blechschmidt teaches us that development takes place when there is a spatial opportunity and a metabolic occasion [19]. He explains the emergence of tissues by defining the fundamental orientation of limiting and inner tissues: limiting tissues are the epithelium on the shore of embryonic fluid spaces or lumens; inner tissues (mesodermal connective tissues) are bounded by the former cells and their basement membranes. All the above is built on an underlying unseen canvas, a Cartesian three-dimensional spatial grid or field which establishes the basic poles of orientation, anterior/ventral, posterior/dorsal, superior/cephalic, and inferior/caudal, and the relative growth–spatial relationships of adjacent tissues and cells. How does a cell “know” where it is? Perhaps the electromagnetic field of the collective atoms and their subatomic particles is the basis of three-dimensional field orientation. This three-dimensional geometry is elastic and bending in time; it is topological. 

Migrating cell ensembles in the developing embryo form their arrangements on a foundation, built of an archaic connective tissue called the interstitium. It is the layer of acellular connective tissue, also known as the extracellular matrix (ECM), or loose areolar tissue. The interstitium is composed of the “inner tissues” of self-organizing structural collagen bundles, which branch and entangle themselves in fractal, spongelike patterns that define serum-filled interstitial spaces, which cannulate because of metabolic contrails. Hyaluronan (HA) has a ubiquitous presence in the interstitial spaces [20]. It can be composed of large, hydrated forms which hold cells together as tissues form, but also small building blocks (created when enzymes known as hyaluronidases break hyaluronan into smaller pieces) serving in a local messaging capacity between cells. This multifunctional molecule is a key component of cellular organization and tissue morphogenesis in metazoans [21]. Over time, the budding tubes of the emerging lactiferous ductal epithelium receive and deposit the products of their metabolic needs in patterns of flow through the labyrinth of the interstitium. In the wake of this metabolic trail, blood vessels emerge. Dike has shown that in vitro endothelial cells grown on a micropatterned substrate that are 10 microns in width and coated with ECM will demonstrate the differentiation and formation of capillary tube-like structures containing a central lumen [22]. Vascular branching *must* follow Adrian Bejan’s Constructal Law of Design: “For a finite size system to persist in time, to live, it must evolve in such a way that it provides easier access to the imposed currents that flow through it” [23]. There is no master architectural plan or DNA code that stipulates this style of branching pattern—given an opportunity, the flow of metabolites will automatically build along the most efficient pattern. In high-metabolic tissues, like growing epithelial parenchyma, this means vascular branching in low fractal dimensions [24]. In the low-metabolic field of the mostly acellular inner tissue, the interstitial circulation of metabolites, morphogens, paracrine substances, and migrating cells (both autologous and invading infectious agents) is based on the ebb and flow of serum through the high fractal dimension, spongelike spaces that exist between the collagen cables of the ECM. The histoarchitecture of the interstitium was first described by Theis et al. in 2018 [20]. In this way, developing organs comprise a parenchymal epithelium supported by connective tissue stromata built on the interstitium which underlies all organ tissues and wraps around all vascular and neuro-cables [25]. The exact differentiation of mesenchymal cells in the developing connective tissue depends on the physical characteristics of the local ECM [26]. We shall return to examine the controlling relationship ECM has on cellular behavior when we look for the beginnings of cancer. 

## 6. The Interstitium as a Body-Wide System 

The interstitium of metazoans most likely was built on a renovated sponge skeletal design, which is made from a collagen-like protein called spongin. The entangled three-dimensional branching of spongin is self-organizing and is produced by specialized cells known as amoebocytes [27]. Sponge cells bind to circular hyaluronic acids like proteoglycans called spongicans [28]. Animal tissues have a spongelike collagen foundation to their connective tissues, with serum and hyaluronan flowing through the open interstitial spaces. It is in this prior art that animal tissues were first invented by nature. The epigenesis of tissues emerges in horizontal laminae, which build vertical depth over time during development. In some ways, this is analogous to 3-D printing, or the rings of a tree trunk, the layers of sedimentary rock, or Piaget’s horizontal and vertical decalage concept, in which intelligence is built over developmental milestones, over space–time. Multicellular organisms are composed of a heterarchy of tissue and organ systems. Whether it is the bottom-up influence of DNA inheritance, which affects the proteome of cells and tissues, or the top-down influence we know as epigenetics, living organisms are complex wholes which must be understood by their interconnectedness.

Animal cells are not amoebas or paramecia that swim through archaic seas. Animal cells live on dry land and must take their sea water with them everywhere they go. They have outer skins which allow for autonomous separation from their environment, and as Claude Bernard first taught, allow for the inner homeostasis of their internal milieu. But all this magic requires organs built of specialized tissues. Also, animal cells do not swim. Animal cells migrate on their ECM scaffolds, like adventurers who free climb on sheer rock walls. 

Donald Ingber has published his findings showing the importance of mechanical forces in morphogenesis and cell differentiation. He explains that the tensegrity-like prestressing of structural ECM proteins outside of the cell connects to the tensioned intracellular cytoskeleton and microtubules, which convey mechanotransduction effects on the nucleus and gene expression. He states, “… mechanical forces generated by living cells are as crucial as genes and chemical signals for the control of embryonical development, morphogenesis and tissue patterning.” [29,30].

**Figure 1:**
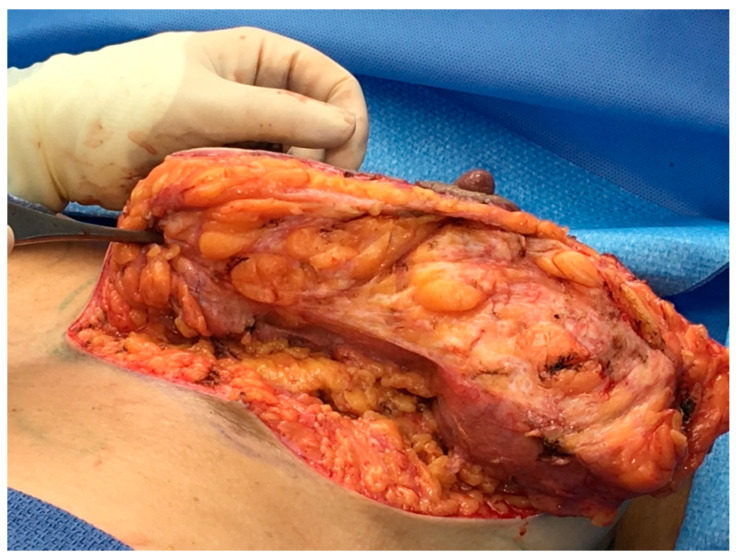

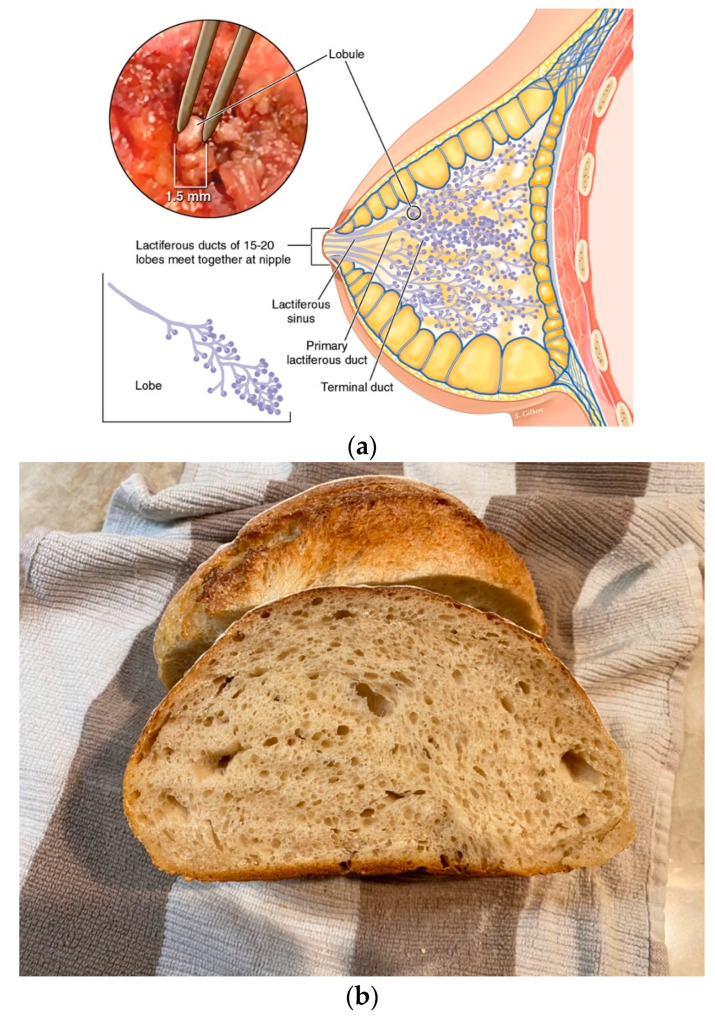
(**a**): The pseudo-capsule of the corpus mammae is composed of compressed collagen which surrounds and contains the breast parenchyma, composed of lactiferous ducts and terminal lobules. (**b**): The crust of a boule of bread is composed of the same ingredients as the bread “sponge”, but in a different structural form at the surface.

**Figure 2:**
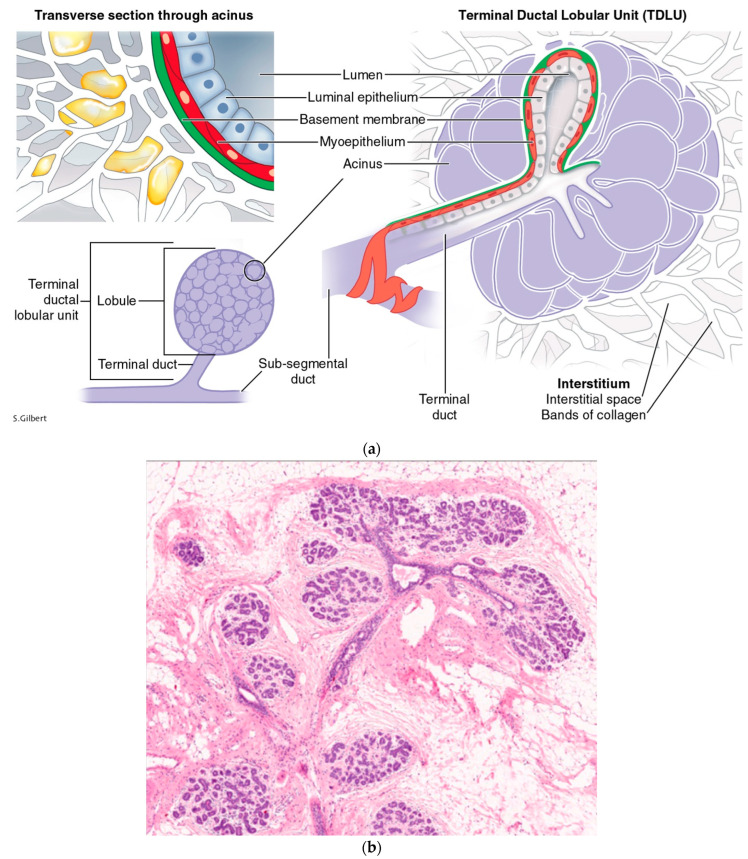
(**a**): The terminal duct lobular unit is the functional unit of the breast epithelial parenchyma. It is embedded in the stromal interstitium. (**b**): Histology of the TDLU of the breast courtesy of Lazlo Tabar, with permission.

**Figure 3:**
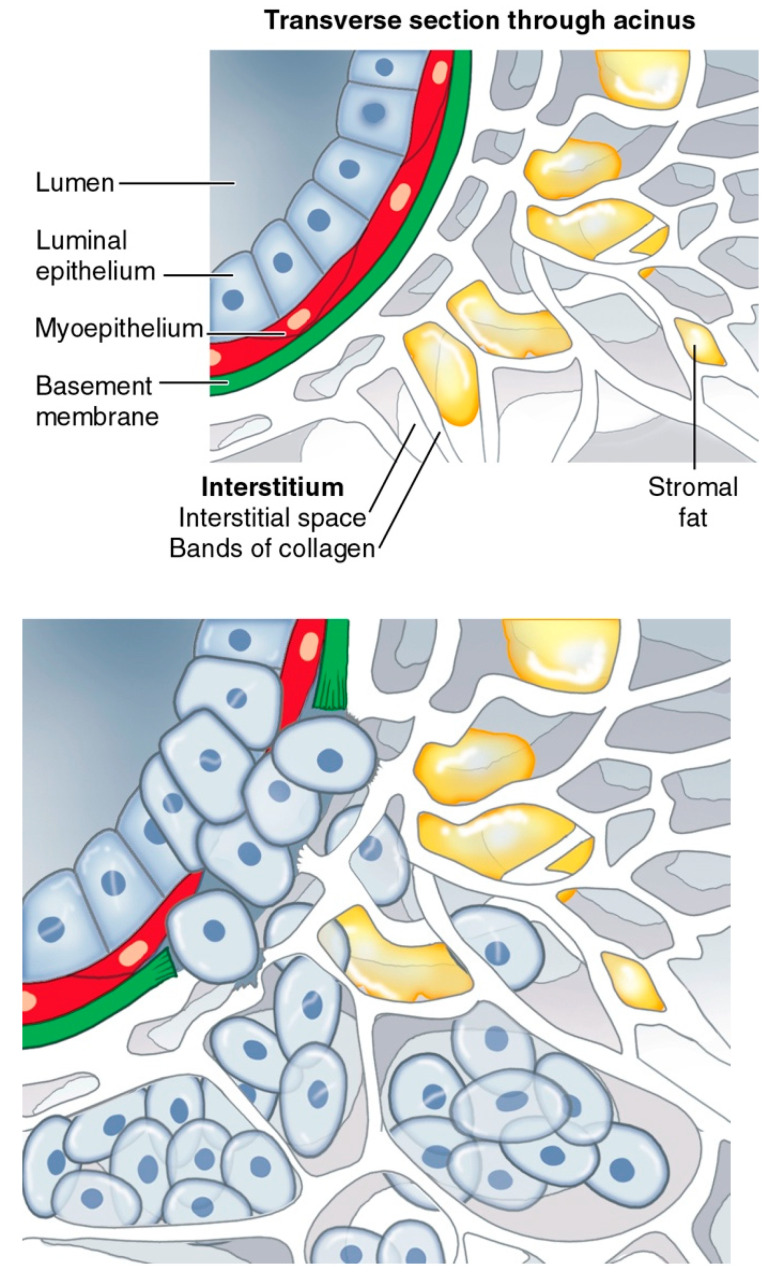
Breakdown of the basement membrane and invasion of the interstitium by neoplastic cells; the cancer tissue environment (CTE).

The second month of human fetal development marks the beginning of the breast as an organ. The fertilized egg has gone through symmetry-breaking cellular divisions that have resulted in the development of the morula. Thomas Meyers calls the infoldings of the conceptus “double bag” maneuvers [31]. The first of these creates the “blastula stage” of the developing embryo. Differential growth rates and their metabolic byproducts physically separate and shape early tissues, which Blechschmidt calls the anlages of the eventual organs. The developing ventral thorax demonstrates a sagittal mammary line to the left and right of the center. On these lines, the human embryo picks a point, between the fourth and fifth ribs, to start its invasion of mesodermal adipose tissues by ectodermal epithelial cells. This process of epithelial branching begins with a remodeling and weakening of the basement membrane by the actions of matrix metalloproteinases [32], which triggers an avalanche of separation in the underlying tensioned, mesodermal interstitium. Ingber likens it to a “run” in a woman’s nylon stocking [30], because the interstitial fibers and filaments are pretensioned in a tensegrity architecture [33]. This tearing crack in the spongy labyrinth of collagen bundles parts the sea of inner tissue, sucking the overlying epithelium into the chasm created in the interstitial ECM, thus making way for the low fractal dimension branching of the breast’s lactiferous ductal system. This is not the time for lobular cell development—that will have to wait many years for the hormonally stimulated beginning of puberty. But even this early coordinated cellular subordination to the common good requires paracrine communication along the local backwater paths of the interstitium. The epithelial invasion of the mesodermal lands pulls with it trailing caissons of vascularity [30]. Branching epithelial ducts become mired in the muck of the interstitium, which eventually halts the momentum of their campaign. The overwhelming flood of ectodermal invagination and branching lactiferous expansion compresses against the interstitial collagen fibers it is invading, and the compressed exterior border becomes known as the pseudo-capsule of the corpus mammae, just like the surface tension of a baking boule of bread forms a crust (Figure 1a,b). The relatively immobile chest wall that is adjacent to the posterior surface of the corpus mammae causes a flat surface, while the more elastic skin/soft tissue envelope on the breast gland’s anterior surface allows for a fluffy morphology, like cumulus clouds. This is the background history of the breast organ, when cancer emerges; in the process of autopoiesis, cellular populations of tissues become mutinous. The end terminal ductal lobular unit (TDLU) (Figure 2a,b) has been called the site of the cancer tumor microenvironment (TME). What is the morphology of this breakdown in breast culture, and should it more correctly be called the cancer tissue environment (CTE) (Figure 3)?

The cancer tissue environment concept acknowledges that the pathologic development of cancer cannot be understood with a reductionist approach. The study of microscopic cancer cell organoids is still not on a large enough scale to understand the fact that cancer is a stochastic emergence of a new relationship between a tissue’s parenchymal cells and its interstitial, stromal matrix. It is not just a mutation in James Watson’s double helix “secret of life”. Life is a more complicated story than the alphabetic code that is used to compose life’s literature. We must search for the meaning that emerges between the words of that literature on life and pathology.

As Carrel stated, “A cell is bound to medium (lymph filling the intra-organic spaces) strictly as nucleus to cytoplasm”. Theise has brought the theoretical “intra-organic space”, this so-called third space, into the real space–time of anatomy. Breast epithelial cells plus the interstitial sponge matrix of structural proteins and the interstitial spaces, filled with serum and glycosaminoglycans like hyaluronan, are the analogs to Carrel’s cytoplasm/nucleus analogy. The tissues of the body do not have parts; they are a tapestry—a seamless dynamic whole. Carrel states with authority that simplifications of biologic complexity, like the mystery of the origins of cancer, are “convenient abstractions” but not reality. It is not enough to study cancer cells in vitro, breaking down the changes in their proteomics brought on by mutation. Instead, we must begin to consider the entire tissue and organ ecosystem.

## 7. The Failure of Organ Culture

**Figure 4:**
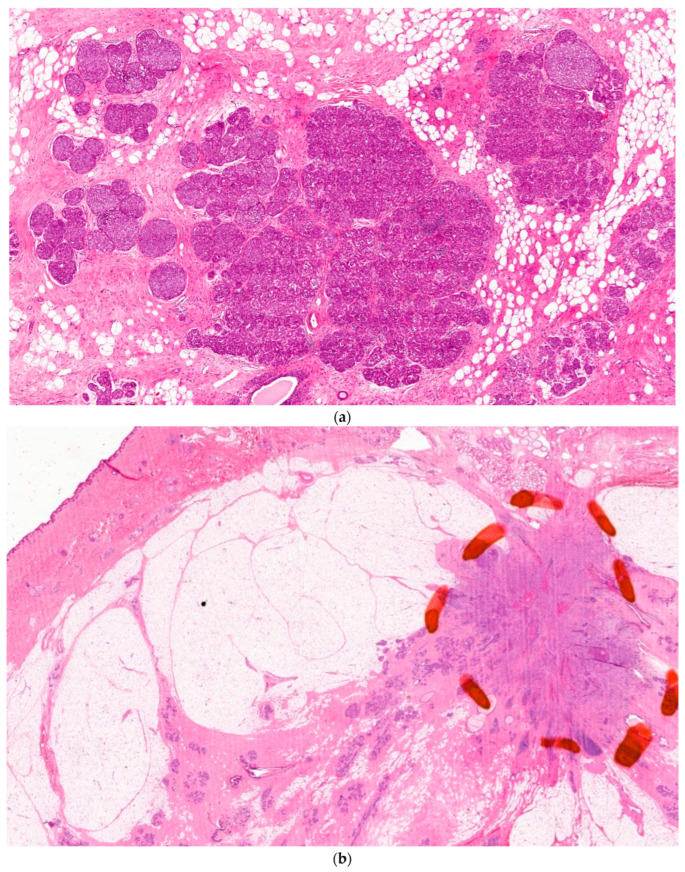
(**a**): Carcinoma in situ of the breast, courtesy of Laszlo Tabar, with permission. (**b**): Invasive breast cancer tumor involving the pseudo-capsule of the corpus mammae, courtesy of Laszlo Tabor, with permission.

It is possible that the snapshot of a breast needle biopsy will catch tissue which exhibits ‘carcinoma in situ’. Abnormal cell behavior in a tissue can be seen on a spectrum of abnormality. In situ neoplasia are insubordinate cellular mitoses, with an intact basement membrane (Figure 4a). Once the basement membrane’s constraining boundary fails, a flood of uncivilized cells enters the adjacent interstitium (Figure 4b). They have crossed the Rubicon. “Cancer cells” do not inhabit adjacent cells in a parasite fashion, the way that some theorists suggest microbes, which are part of the tumor microbiome in the interstitium, become parasites in tumor cells [6]. Therefore, by definition, they inhabit *interstitial* space. Invasive cancer is “looking” for room to grow within the expandable interstitial space, defined by the bundles of collagen it infiltrates. The interstitium is characterized by its dynamic ability to enlarge dramatically because of inflammation, binding more volumes of water to hyaluronan, thus setting the stage for the required space for tumors to grow [21].

These multiplying epithelial cells lose their connection to each other when connections called E-cadherins stop being expressed, severing the connections from epithelial cells to basement membranes, hyaluronan, and fibronectin. Through the failures of desmosomes, gap junctions, and tight and anchoring junctions, epithelial cells separate, lose their polarity and cross the dissolving basement membrane, and crawl into the interstitium. This withdrawal from the organ community unhinges their mechanical connections to each other and the local extracellular proteins. Dedifferentiation and migration are hallmarks of the so-called epithelial to mesenchymal transition, or EMT for short. Spicer and Tien’s dramatic conclusion to their review paper, “Hyaluronan and Morphogenesis” [21], states, “HA can be thought of as both the beginning and the end, an alpha and omega, of many major developmental or morphogenetic processes… to effect changes in cellular shape, form, and function, such as EMT, as well as changes in cellular behavior, such as cell proliferation and cell migration”. Once they arrive in adequate tissue tumor environments, like the interstitium, they revert to epithelial cells in a process termed the mesenchymal to epithelial transition (MET) and begin to grow, enlarging tumors. Perhaps cancer’s in statu nascendi begins with a failure of tissue maintenance and hyaluronan metabolism.

Tumor cells frequently are found to have hybrid states, exhibiting both epithelial and mesenchymal traits [34]. These tumors are frequently highly aggressive tumors, such as triple-negative breast cancers [35].

**Figure 5:**
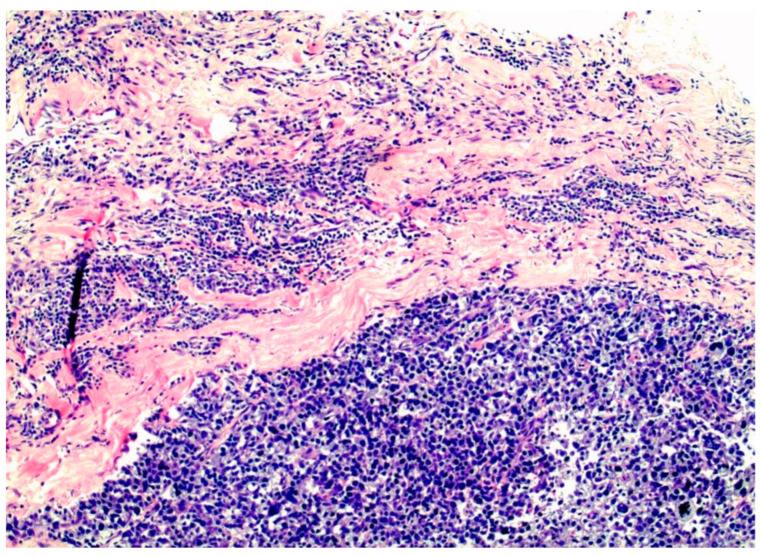
Triple-negative, basal-like breast cancer filling the interstitial spaces between the pink collagen bundles of the interstitium.

The isolation from their tissue confraternity leads to the change to a spherical cellular shape, and the resulting dedifferentiation. The failure of following apoptosis programs and immune surveillance allows instead for a malignant remodeling of the interstitial ECM (Figure 5).

The breakdown in the coordinated attachment of the epithelial lining is like a dancing couple getting out of step. The cells no longer are following a collective choreography but enter a mosh pit. The timing, rhythm, and movement of cell growth breaks all the rules of tissue culture. A “cancer cell” revolts against subordination to the collective good of tissues, and according to Sonnenschein and Soto, reverts to its natural state: constant growth, division, and migration. These authors have long argued for a more holistic understanding of cancer as it relates to the whole organism through the disciplines of systems biology and morphogenesis controlled by self-organization [36].

The high-metabolic growing tumor cell mass demands more blood flow but there is no longer the branching epithelium of lactiferous ducts, with its low fractal pattern, pushing out ahead of the following vasculature [30]. The trails of cancer metabolites flow haphazardly in the high fractal dimension pattern of the interstitial spaces, and thus the emerging tumor vascularity is different from normal breast tissue vascularity [37]. The instability of a drop in the economic output of tumor metabolism opens the door to a metabolic coup, switching from oxidative phosphorylation to glycolysis [38,39]. When the delivery of oxygen and nutrients no longer keep pace with the rate of growth of the tumor, central necrosis takes place. Undifferentiated cells with their high mitotic rate then begin to look for greener pastures to emigrate to. This is the likely cause of tumor macrophage activity that senses and changes the interstitial milieu, thus triggering the epithelial to mesenchymal transition (EMT) and metastasis [40]. Metastatic cells may find walls of resistance and unreceptive environments in the ECM of distant tissues, but they may find receptive, liberal shores to immigrate to and build tumor colonies. If Bejan is right, the constructal law will say that eventually the flow of the growing tumor economy cannot be sustained [23]. The organism racked with growing metastatic colonies will not persist over time.

Perhaps it is time we contemplate the dynamic relationships between autopoietic tissue components, interstitial hyaluronan, the breast parenchymal epithelium, and the state of an organ’s humors, not just a “cancer cell’s” DNA mutations. This should change the questions we formulate about cancer, the observations we make, and ultimately the strategies we produce in this struggle.

The questions of this old song should inspire all cancer research:

“Oh, very young

What will you leave us this time?

You’re only dancing on this Earth for a short while

…

And though you want to last forever

You know you never will

You know you never will

And the goodbye makes the journey harder still

Oh, very young

What will you leave us this time?

Your only dancing on this Earth for a short while

Oh very young

What will you leave us this time?”—Cat Stevens

## 8. Conclusions

The breakdown in the *cell culture* of tissues in a specific organ is the beginning of cancer. Here, too, we hope is the secret to the end of cancer. To overcome cancer, the “culture” of organs must be understood and conserved.

## Data Availability

No new data were created or analyzed in the creation of this essay. Data sharing is not applicable to this article.
